# Cortisol modulates inflammatory responses in LPS-stimulated RAW264.7 cells via the NF-κB and MAPK pathways

**DOI:** 10.1186/s12917-018-1360-0

**Published:** 2018-01-30

**Authors:** Junsheng Dong, Jianji Li, Luying Cui, Yefan Wang, Jiaqi Lin, Yang Qu, Heng Wang

**Affiliations:** 1 College of Veterinary Medicine, Yangzhou University, Yangzhou, Jiangsu 225009 China; 2 Jiangsu Co-innovation Center for the Prevention and Control of Important Animal Infectious Diseases and Zoonoses, Yangzhou, Jiangsu 225009 China

**Keywords:** Macrophage, Cortisol, Anti-inflammatory, LPS, NF-κB, MAPKs

## Abstract

**Background:**

The uteruses of most dairy cattle are easily infected by bacteria, especially gram-negative bacteria, following parturition. Macrophages are important cells of the immune system and play a critical role in the inflammatory response. In addition, cortisol levels become significantly increased due to the stress of parturition in dairy cattle, and cortisol is among the most widely used and effective therapies for many inflammatory diseases. In this study, we assessed the anti-inflammatory effects and potential molecular mechanisms of cortisol using a Lipopolysaccharide (LPS)-induced RAW264.7 macrophage cell line.

**Results:**

Cortisol significantly suppressed the production of prostaglandin E_2_ (PGE_2_) and decreased the gene and protein expression of inducible NO synthase (iNOS) and cyclooxygenase-2 (COX-2) in a dose-dependent manner. Moreover, cortisol inhibited the mRNA expression of pro-inflammatory cytokines including tumor necrosis factor alpha (TNFα), interleukin-1β (IL-1β), and interleukin-6 (IL-6) and decreased IL-1β secretion in an LPS-treated RAW264.7 macrophage cell line. Moreover, we found that cortisol suppressed nuclear factor-kappa B (NF-κB) signaling in RAW264.7 macrophages stimulated with LPS. This suppression was mediated by the inhibition of IκBα degradation and NF-κB p65 phosphorylation. In addition, cortisol also suppressed the phosphorylation of mitogen-activated protein kinases (MAPK) such as extracellular signal-regulated kinase (ERK1/2), p38 MAPK, and c-Jun N-terminal kinase/stress-activated protein kinase (JNK).

**Conclusions:**

These results suggest that high cortisol levels can attenuate LPS-induced inflammatory responses in the RAW264.7 macrophage cell line by regulating the NF-κB and MAPK signaling pathways.

## Background

Postpartum uterine infection and inflammation are the primary causes of reproductive failure in dairy cows [1, 2]. Almost all cows are susceptible to bacterial infection at the openings of anatomic barriers including the vulva, vagina, and cervix. Lipopolysaccharide (LPS) is the most common pathogenic endotoxin component in the outer membrane of gram-negative bacteria and can disturb the balance between immunity and inflammatory responses [3]. Inflammation is a major risk factor for many diseases, and macrophages are important immune cells that act as the first line of defense against invading agents (bacteria, viruses, and fungi) [4, 5]. During inflammation, macrophages produce excessive amounts of inflammatory mediators such as prostaglandin E_2_ (PGE_2_), inducible nitric oxide synthase (iNOS) and cyclooxygenase-2 (COX-2) and pro-inflammatory cytokines including interleukin-1β (IL-1β), IL-6, and tumor necrosis factor-alpha (TNFα) [6]. Moreover, iNOS and COX-2 are believed to be the most important inflammatory mediators [7]. Overproduction of these mediators can be harmful to animal organs.

Nuclear factor kappa-B (NF-κB) and mitogen-activated protein kinase (MAPK) are important signaling molecules in the Toll-like receptor (TLR) pathway [8, 9]. NF-κB plays an important role in regulating the inflammatory responses by increasing the expression of inflammatory mediators and pro-inflammatory cytokines such as PGE_2_, iNOS, COX-2, IL-1β, IL-6 and TNFα [10]. Under unstimulated conditions, heterodimers of NF-κB components, mainly p50/p65, remain in the cytoplasm in an inactive form due to linkage to the inhibitor of κB (IκB) protein. However, when induced by LPS, NF-κB (p50/p65) is released through the phosphorylation and degradation of IκB. As a result, NF-κB p65, which is believed to play a central role in inflammation, enters the nucleus and encodes various cytokines and chemokines [11–13]. The MAPKs represent a specific class of serine/threonine kinases that respond to extracellular signals, including extracellular signal-regulated kinase 1/2 (ERK1/2), p38, and c-Jun NH2-terminal kinase (JNK). Similar to NF-κB, the MAPK signaling pathways are involved in LPS-induced iNOS and COX-2 expression in activated macrophages [14]. Even more importantly, MAPKs play essential roles in the activation of NF-κB [15]. Therefore, inhibition of the NF-κB and MAPK pathways may be a potential therapeutic approach to inflammatory injury.

Dairy cows have high levels of cortisol due to many kinds of stress during the perinatal period, such as pregnancy, labor, and lactation [16–18]. Furthermore, cortisol effectively protects immune cells from excessive inflammation [19]. However, neither the anti-inflammatory activity of cortisol on macrophages nor the mechanism of this process has been reported.

In this study, we demonstrated the anti-inflammatory properties of cortisol on LPS-induced inflammation injury in the RAW264.7 macrophage cell line. We further investigated the ability of cortisol to inhibit the activation of NF-κB and mitogen-activated protein kinases (MAPKs) to clarify the mechanism of its anti-inflammatory effects. This study may reveal a vital role for endogenous glucocorticoids and the underlying mechanism of glucocorticoid-mediated anti-inflammatory activity in the postpartum cow uterus, thus proposing a scientific basis for the prevention and treatment of endometritis in dairy cattle.

## Methods

### Reagents

Dulbecco’s modified Eagle’s medium (DMEM), fetal bovine serum (FBS), and other tissue culture reagents were purchased from Gibco BRL Co. (Grand Island, NY, USA). Cortisol (H0888) and LPS (*Escherichia coli* 0111:B4) were purchased from Sigma (St. Louis, MO, USA). The Cell-Counting Kit-8 (CCK-8) reagents were obtained from Dojindo Molecular Technologies, Inc. (Kumamoto, Japan). Enzyme-linked immunosorbent assay (ELISA) kits for PGE_2_, IL-1β, IL-6, and TNFα were purchased from R & D Systems, Inc. (Minneapolis, MN, USA). β-actin, iNOS, COX-2, ERK1/2, phospho-ERK1/2, p38, phospho-p38, JNK, phospho-JNK, NF-κB p65, phospho-NF-κB p65, IκBα and phospho-IκBα antibodies were purchased from Cell Signaling Technology (Boston, MA, USA).

### Cell culture and viability assays

The RAW264.7 macrophage cell line was obtained from the American Type Culture Collection (ATCC, MD, US). The cells were cultured at 37 °C in DMEM supplemented with 2 mM glutamine, 100 U/mL penicillin, 100 μg/mL streptomycin and 10% fetal bovine serum (FBS) in a 5% CO_2_ environment [20]. To evaluate cell viability, RAW264.7 cells (5 × 10^3^ cells/well) were seeded in 96-well plates and incubated for 18 h before experimental interventions. The cells were then treated with several concentrations of cortisol for 24 h. Ten microliters of the CCK-8 solution was added to each well, and the plate was incubated at 37 °C for 2 h. The optical density was then read at 450 nm using a microplate reader (Tecan, Austria).

### PGE_2_, IL-1β, IL-6, and TNFα assays

RAW 264.7 cells were seeded in 12-well plates (5 × 10^5^ cells/mL) and incubated at 37 °C for 18 h. The cells were co-treated with cortisol (5, 15 and 30 ng/mL) and LPS (1 μg/mL) for 6, 12 and 24 h. Supernatant levels of PGE_2_, IL-1β, IL-6, and TNFα were measured by ELISA according to the manufacturer’s instructions.

### RNA extraction and real-time quantitative reverse transcription PCR

RAW 264.7 macrophages were treated with 1 μg/mL LPS in the presence or absence of cortisol (0, 5, 15 and 30 ng/mL). After 6-, 12- and 24-h incubation periods, total RNA was isolated from RAW 264.7 macrophages according to the manufacturer’s instructions using Trizol reagent (Invitrogen, US). The extracted RNA was quantified using a Nanodrop 2000 spectrophotometer (Thermo, USA). The RNA (900 ng) was then converted to cDNA as previously described [21]. The PCR contained 10 μL SYBR Green PCR mix, 0.5 μL each primer, and 1 μL cDNA template in a final reaction volume of 20 μL (Takara, Japan). The real-time PCR cycling conditions were 95 °C for 2 min, 40 cycles of 95 °C for 10 s, 60 °C for 30 s, and 72 °C for 30 s using a CFX connect real-time PCR system (BIO-RAD, US). The rat β-actin primers were used as the endogenous control. Relative gene expression was calculated using the comparative Ct method (2^-△△Ct^) as previously described [22]. The primer sequences used in this study are presented in Table 1.

**Table 1:** Primer sequences used for qRT-PCR amplification

Gene	Forward primer	Reverse primer	Accession number	Product size(bp)
β-actin	TGCTGTCCCTGTATGCCTCT	TTTGATGTCACGCACGATTT	NM_031144.3	224
IL-1β	ACCTGTGTCTTTCCCGTGG	TCATCTCGGAGCCTGTAGTG	NM_031512.2	159
TNFα	GCCTCCCTCTCATCAGTTCTA	GGCAGCCTTGTCCCTTG	NM_012675.3	246
IL-6	AGTTGTGCAATGGCAATTCTGA	AGGACTCTGGCTTTGTCTTTCT	NM_012589.2	223
iNOS	TTCCAGAATCCCTGGACAAG	TGGTCAAACTCTTGGGGTTC	NM_012611.3	180
COX-2	AGAAGGAAATGGCTGCAGAA	GCTCGGCTTCCAGTATTGAG	NM_017232.3	194

### Western blot analysis

RAW264.7 macrophages were stimulated with LPS alone or together with cortisol as described above. The total proteins were extracted, and protein concentrations were determined using a bicinchoninic acid (BCA) protein assay kit (BioChain, US). Total proteins were separated by sodium dodecyl sulfate-polyacrylamide gel electrophoresis (SDS-PAGE) and transferred to polyvinylidene difluoride (PVDF) membranes (Millipore, Germany). The membranes were immunoblotted with primary antibodies specific for iNOS, COX-2, NF-κB p65, phospho-NF-κB p65, IκBα, phospho-IκBα, p-ERK1/2, ERK1/2, p-p38, p38, p-JNK, JNK, and β-actin at 4 °C overnight and then incubated with HRP-conjugated secondary antibodies (CST, US) at room temperature for 1 h. The blots were washed with PBS-T, and the proteins of interest were detected using a chemiluminescence (ECL) assay according to the manufacturer’s instructions.

### Statistical analysis

Each experiment was repeated at least three times, and all data are expressed as means ± standard error of the mean (SEM) for the number of experiments. Statistically significant differences throughout this study were calculated by one-way ANOVA followed by Dunnett’s test (SPSS 17.0 software). A two-sided *p*-value less than 0.05 was considered statistically significant.

## Results

### Effect of cortisol on RAW264.7 macrophage viability

**Fig. 1:**
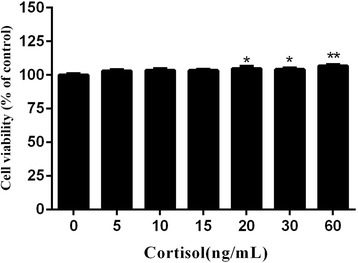
Effects of different concentrations of cortisol on RAW264.7 cell viability as measured by the CCK-8 assay. The data shown are means ± SEM (*n* = 6). *p < 0.05 and **p < 0.01 vs. control group

The effect of cortisol on the RAW264.7 macrophage cell line viability was assessed using a CCK-8 assay. As shown in Fig. 1, cortisol did not affect the viability of the RAW 264.7 cells at concentrations from 5 to 15 ng/mL, but it did alter cell growth at 20 to 60 ng/mL. Therefore, cortisol concentrations of 5, 15, and 30 ng/mL were selected for further investigation.

### Cortisol modulation of extracellular PGE_2_, TNFα, IL-1β, and IL-6 production in LPS-induced RAW264.7 macrophages

**Fig. 2:**
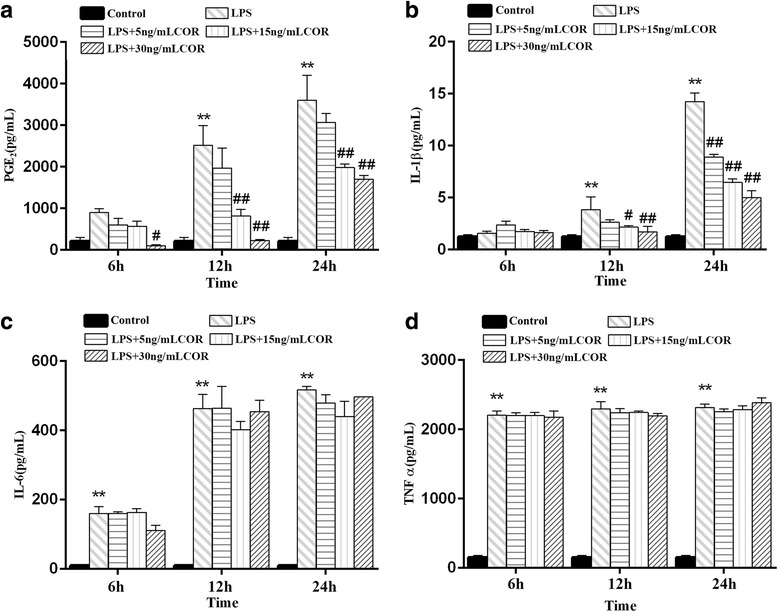
Effect of cortisol on PGE_2_ and cytokine production in LPS-stimulated RAW 264.7 macrophages. RAW264.7 cells were co-treated with cortisol (0, 5, 15 and 30 ng/mL) and LPS (1 μg/mL) for 0, 6, 12, and 24 h. Levels of PGE_2_ (**a**), IL-1β (**b**), IL-6 (**c**), and TNFα (**d**) in culture supernatants were measured by ELISA. The data presented are the means±SEM. ** *p* < 0.01 vs. the control group; # *p* < 0.05, ## *p* < 0.01 vs. the LPS group

To investigate the inhibitory effects of cortisol on the extracellular production of inflammatory mediators and pro-inflammatory cytokines including PGE_2_, TNFα, IL-1β, and IL-6 by LPS-induced RAW264.7 macrophages, cytokine-specific ELISAs were used to determine the levels of each molecule in RAW264.7 culture supernatants. As depicted in Fig. 2a, the PGE_2_ concentration in the culture medium of the LPS-treated group was significantly (*p* < 0.01) increased compared with the control group at 12 and 24 h. However, co-incubation with cortisol significantly (*p* < 0.05) suppressed this increased production in a dose-dependent manner. The expression levels of IL-1β, IL-6, and TNFα induced by LPS were significantly upregulated at the indicated time points (*p* < 0.01). However, cortisol significantly suppressed the extracellular levels of IL-1β when compared with the LPS treated group in a dose-dependent manner (Fig. 2b). The levels of TNFα and IL-6 were not affected by cortisol treatment (Fig. 2c and d).

### Effects of cortisol on the protein and mRNA expression levels of iNOS and COX-2 in LPS-stimulated RAW264.7 macrophages

**Fig. 3:**
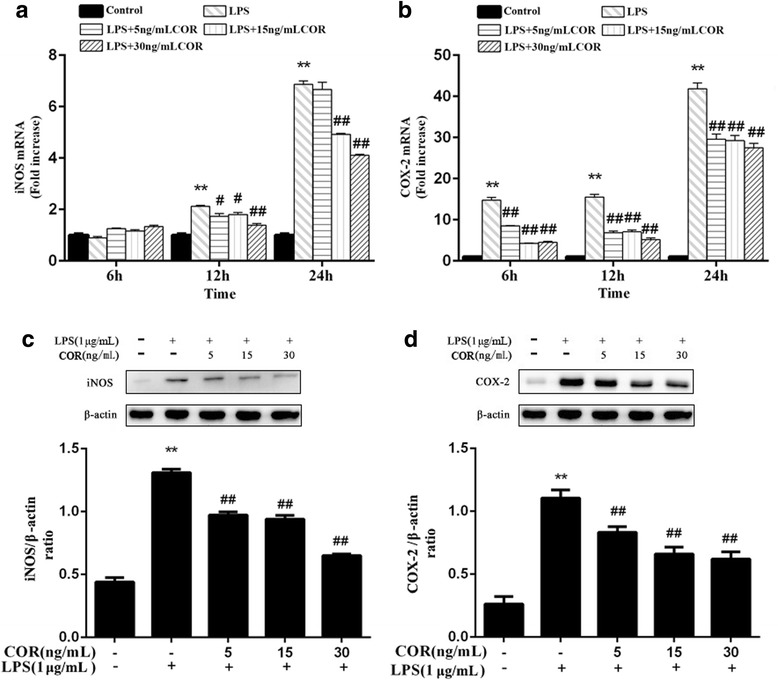
Effects of cortisol on the mRNA and protein expression levels of iNOS and COX-2 in LPS-stimulated RAW264.7 cells. **a** and **b** Cells were co-treated with cortisol (5,15 and 30 ng/mL) and LPS (1 μg/mL) for 0, 6, 12, and 24 h. RNA was isolated and analyzed by RT-PCR. **c** and **d** Cells were co-treated with cortisol (5, 15 and 30 ng/mL) and LPS (1 μg/mL) for 24 h. Total proteins were isolated and analyzed by Western blot. The data presented are the means±SEM. ** *p* < 0.01 vs. the control group; # *p* < 0.05, ## *p* < 0.01 vs. the LPS group

Since COX-2 and iNOS are enzymes for PGE_2_ and NO synthesis, we further investigated the inhibitory effects of cortisol treatment on COX-2 and iNOS expression using Western blotting and RT-PCR, respectively. As shown in Fig. 3a, the mRNA expression level of iNOS dramatically (*p* < 0.01) increased following stimulation of macrophages with LPS at 12 and 24 h. The mRNA expression levels of iNOS in the experimental groups were down-regulated by cortisol treatment at all concentrations (*p* < 0.05). Similarly, the COX-2 mRNA levels were significantly increased by stimulation of macrophages with LPS at 6, 12, and 24 h (Fig. 3b). In addition, COX-2 mRNA levels were also inhibited by cortisol in a dose-dependent manner.

The protein expression levels of iNOS and COX-2 were significantly (*p* < 0.01) upregulated by stimulation of macrophages with LPS at 24 h. However, these effects were markedly (*p* < 0.01) inhibited by cortisol treatment in a dose-dependent manner (Fig. 3c and d).

### Inhibitory effect of cortisol on LPS-induced TNFα, IL-1β and IL-6 mRNA expression

**Fig. 4:**
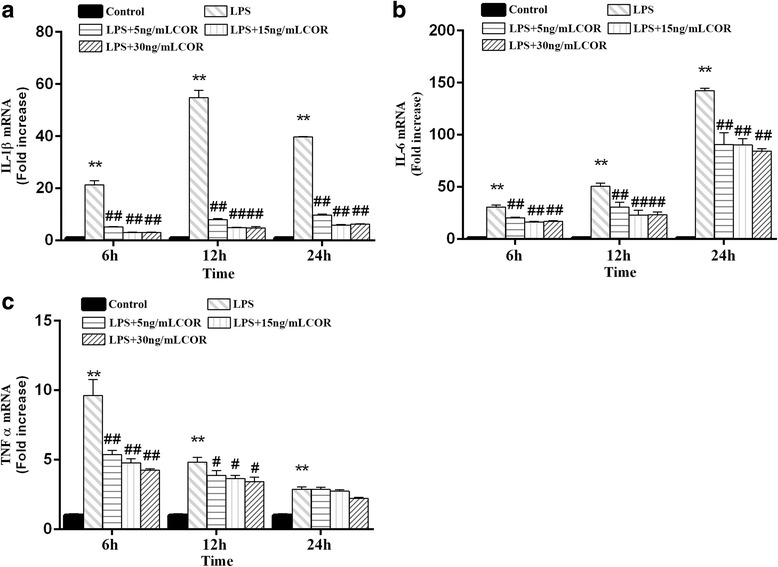
Effects of cortisol on IL-1β (**a**), IL-6 (**b**) and TNFα (**c**) mRNA expression in LPS-stimulated RAW264.7 cells. RAW264.7 cells were co-treated with cortisol (5, 15 and 30 ng/mL) and LPS (1 μg/mL) for 0, 6, 12, and 24 h. RNA was isolated and analyzed by RT-PCR. The data presented are the means±SEM. **p < 0.01 vs. the control group; # *p* < 0.05, ## *p* < 0.01 vs. the LPS group

To determine the protective effect of cortisol on RAW264.7 macrophage inflammatory responses induced by LPS, we examined the mRNA expression levels of TNFα, IL-1β and IL-6 by RT-PCR. As shown in Fig. 4, the expression of TNFα, IL-1β and IL-6 induced by LPS was significantly upregulated at the indicated time points, whereas dose-dependent reductions in LPS-stimulated TNFα, IL-1β, and IL-6 mRNA expression levels were observed in macrophages after co-incubation with cortisol (*p* < 0.01).

### Effects of cortisol on NF-κB activation in LPS-stimulated RAW264.7 macrophages

**Fig. 5:**
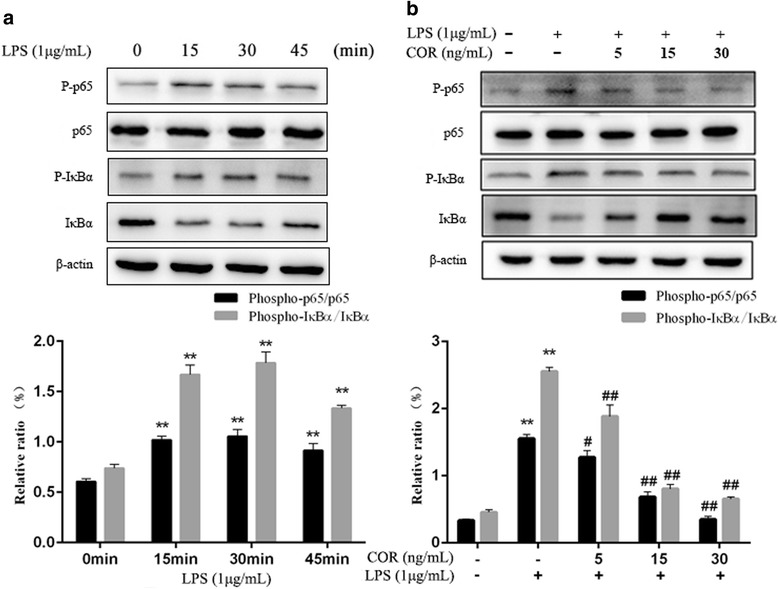
Inhibitory effects of cortisol on LPS-stimulated NF-κB p65 and IκBα phosphorylation in RAW 264.7 cells. **a** Cells were stimulated with LPS (1 μg/mL) alone for 0, 15, 30 and 45 min. **b** Cells were co-treated with cortisol (5, 15 and 30 ng/mL) and LPS (1 μg/mL) for 30 min. Total proteins were isolated and subjected to Western blotting. The data presented are the means±SEM. ** *p* < 0.01 vs. the control group; # *p* < 0.05, ## *p* < 0.01 vs. the LPS group

NF-κB is an important transcription factor that regulates the expression of most pro-inflammatory cytokines, as well as the levels of iNOS, COX-2, and PGE_2_. We investigated the critical proteins of this signaling pathway by Western blotting to determine the effect of cortisol on the NF-κB activity. As shown in Fig. 5, significant (*p* < 0.01) degradation of IκBα and increased expression of p-IκBα and p-p65 were observed in the cells following LPS exposure for 30 min, which indicated increased NF-κB activity. However, the degradation of IκBα and phosphorylation of IκBα and p65 were decreased after 45 min. Cortisol significantly inhibited the LPS-induced phosphorylation of p65, as well as phosphorylation and degradation of IκBα, in a dose-dependent manner. The data showed that the NF-κB activity in RAW264.7 macrophages induced by LPS was significantly (*p* < 0.01) inhibited by cortisol.

### Effects of cortisol on the phosphorylation of MAPKs in LPS-stimulated RAW264.7 macrophages

**Fig. 6:**
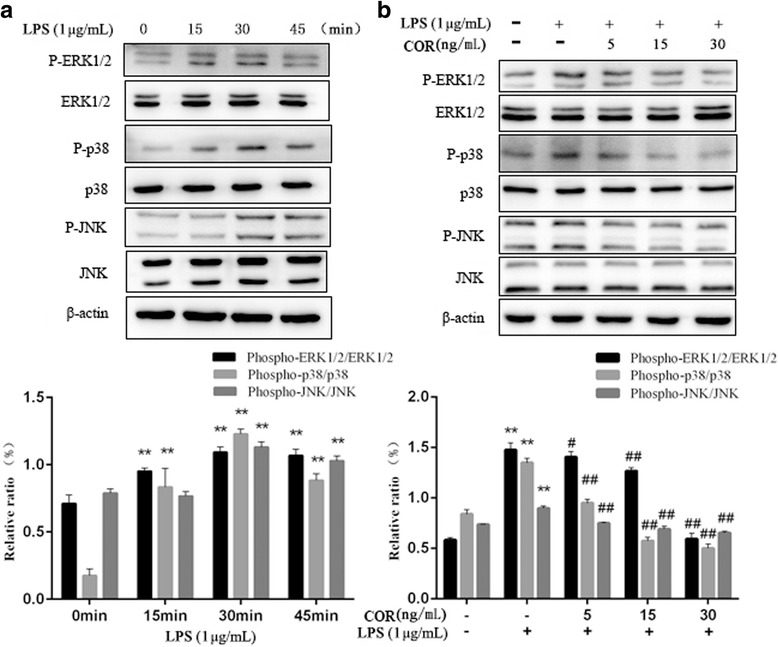
Inhibitory effects of cortisol on MAPK phosphorylation in RAW264.7 macrophages. **a** Cells were stimulated with LPS (1 μg/mL) alone for 0, 15, 30 and 45 min. **b** Cells were co-treated with cortisol (5, 15 and 30 ng/mL) and LPS (1 μg/mL) for 30 min. Total proteins were isolated and subjected to Western blotting. The data presented are the means±SEM. ** *p* < 0.01 vs. the control group; # *p* < 0.05, ## *p* < 0.01 vs. the LPS group

MAPKs play important roles in the regulation of various physiological processes [23]. To determine the effect of cortisol on the MAPK pathway, we investigated the critical proteins of this signaling pathway by Western blot. The phosphorylation levels of ERK1/2, JNK, and p38 MAPK were significantly (*p* < 0.01) increased after the cells were treated with LPS for 30 min (Fig. 6a). However, the levels of phosphorylation were decreased after 45 min. Cortisol significantly (*p* < 0.01) inhibited the LPS-induced phosphorylation of ERK1/2, JNK, and p38 MAPK in a dose-dependent manner (Fig. 6b).

## Discussion

In this study, we examined the anti-inflammatory activities of cortisol in LPS-induced RAW264.7 cells. Cortisol significantly inhibited the expression levels of inflammatory mediators and pro-inflammatory cytokines (Figs. 3 and 4). Moreover, the NF-κB and MAPK activities in LPS-induced RAW264.7 macrophages were obviously alleviated by cortisol (Figs. 5 and 6).

After parturition, dairy cows are more susceptible to endometritis, which is the primary cause of reproductive failure [2]. If not treated in a timely manner, the inflammatory response generates more serious consequences that lead to endometritis and even purulent uterine inflammation. Cortisol is a major regulator of inflammation and may play a role in preventing inflammation in the body [24]. Perinatal stress triggers the release of corticotropin-releasing hormone (CRH) from the hypothalamus, which acts on the pituitary to release adrenocorticotropin hormone (ACTH) and subsequently on the adrenal glands to release cortisol into blood circulation. In addition, cortisol production peaks due to the stress of parturition [16, 25].

A rapid inflammatory response is produced in LPS-stimulated RAW264.7 macrophages, which could release a large number of pro-inflammatory cytokines (TNFα, IL-1β, and IL-6) and inflammatory mediators (PGE_2_, COX-2 and iNOS) [6]. This is beneficial to attract circulating immune effector cells, such as neutrophils, to fight infection [26], but excessive inflammatory responses can injure tissues and organs. Therefore, the expression of inflammatory mediators and pro-inflammatory cytokines needs to be tightly regulated during an inflammatory response [27, 28]. In this study, we demonstrated the protective effect of cortisol against LPS-induced inflammation injury in the RAW264.7 macrophage cell line. The results showed that the gene expression and production of TNFα, IL-1β, and IL-6 were significantly increased in RAW264.7 macrophages stimulated with LPS, which induced a drastic inflammatory response. As expected, cortisol effectively inhibited the mRNA expression levels of IL-1β, IL-6, and TNFα in a dose-dependent manner, which protected macrophages from LPS-induced inflammation injury. Interestingly, there were inconsistencies between the mRNA expression and secretory protein levels of IL-6 and TNFα, which may be related to the translational regulation or the cytoplasm storage of these molecules. However, the specific mechanism behind this phenomenon requires further investigation.

NO and PGE_2_ are important inflammatory mediators that result in serious inflammatory diseases. iNOS catalyzes the oxidative deamination of L-arginine and ultimately leads to significant nitric oxide (NO) production. Similarly, COX-2 is a key enzyme involved in the biosynthesis of prostaglandin E_2_ (PGE_2_). Thus, reducing the levels of iNOS and COX-2 would be an effective strategy for suppressing inflammatory responses. Our study demonstrated that cortisol inhibited extracellular production of PGE_2_ in a dose-dependent manner at all time points. Moreover, cortisol inhibited LPS-induced iNOS and COX-2 mRNA and protein levels in a dose-dependent manner. These results indicated that cortisol could effectively inhibit the LPS-induced inflammatory response.

NF-κB is an important regulatory transcription factor that plays a critical role in regulating the expression of iNOS, COX-2 and pro-inflammatory cytokines such as TNFα, IL-1β and IL-6. Once activated by LPS, phosphorylation of IκBα is strongly enhanced (IκBα is the principal inhibitory protein of NF-κB), which leads to the rapid proteasomal degradation [29, 30] of IκBα. Nuclear factor κB dimers (p50:p65) are released and phosphorylated, which quickly enter the nucleus and bind specifically to defined DNA sequences to promote target gene expression [11, 12, 31]. Thus, we investigated the inhibitory effect of cortisol on LPS-induced NF-κB activation. The present study showed that the degradation of IκBα and the phosphorylation of IκBα and p65 were significantly increased after stimulation with LPS for 30 min, which suggested obviously increased NF-κB activation. However, the degradation of IκBα and the phosphorylation of IκBα and p65 were reduced after 45 min due to the synthesis and secretion of IκBα, which was consistent with the results of previous studies [32, 33]. Our results suggested that cortisol could reduce the degradation of IκBα and phosphorylation of IκB α and p65 at 30 min in a dose-dependent manner, demonstrating a significant inhibitory effect on NF-κB activity. Thus, cortisol could inhibit the inflammatory mediator and pro-inflammatory cytokine expression by downregulating the NF-κB pathway. Ultimately, inflammatory injury in the LPS-induced RAW264.7 macrophage cell line was significantly weakened.

In addition to NF-κB, much evidence has shown that the MAPK pathway also plays an important role in the inflammatory response. The MAPK family includes ERK1/2, JNK, and p38, which play a critical role in the transcriptional regulation of the LPS-induced expression of iNOS and COX-2 [34]. Moreover, MAPKs are known as upstream activators of NF-κB [35], as demonstrated by the inhibition of NF-κB transcriptional activation by specific MAPK inhibitors [36]. In the present study, the phosphorylation of ERK1/2, JNK, and p38 was significantly increased after LPS stimulation of RAW 264.7 macrophages, indicating that LPS activated the MAPK signaling pathway in the RAW264.7 macrophage cell line. Cortisol treatment obviously inhibited the phosphorylation of ERK1/2, JNK, and p38. These results suggested that the anti-inflammatory effects of cortisol are related to inhibition of MAPK phosphorylation in LPS-induced RAW 264.7 cells.

## Conclusions

In conclusion, the current study clearly demonstrated the protective effect of cortisol on LPS-induced inflammation injury in the RAW264.7 macrophage cell line. Cortisol inhibited LPS-induced iNOS and COX-2 expression, as well as PGE_2_ production, in the macrophages. Equally, it also inhibited the expression of pro-inflammatory cytokines, including IL-1β, IL-6 and TNFα. The anti-inflammatory effect of cortisol on macrophages is mediated through inhibition of the NF-κB and MAPK signaling pathways.

## Abbreviations

COR: Cortisol
ELISA: Enzyme-linked immunosorbent assay
LPS: Lipopolysaccharide
MAPK: Mitogen-activated protein kinase
NF-κB: Nuclear factor kappa-light-chain-enhancer of activated B cells
qRT-PCR: Real-time quantitative reverse transcription PCR
